# Does the low-carbon pilot policy improve urban economic resilience? Evidence from China

**DOI:** 10.1371/journal.pone.0284740

**Published:** 2023-04-21

**Authors:** Zhiyu Liu

**Affiliations:** School of Finance, Southwestern University of Finance and Economics, Chengdu, Sichuan, China; East China University of Science and Technology, CHINA

## Abstract

Identifying the relationship between carbon neutrality initiatives and its economic impact is crucial in evaluating the cost of low-carbon transition for policy makers. In this paper, a theoretical model is built to discuss the effects of the low-carbon pilot policy in China on urban economic resilience and an empirical test is conducted to examine the relationship using the Heckman two stage model and a panel data of 277 cities from 2004 to 2020. The results show that low-carbon pilot policy significantly enhanced urban economic resilience and the stimulating effect is mainly achieved by motivating technology innovations. In addition, further analysis indicates that low-carbon pilot policy has a more pronounced effect on improving urban economic resilience of cities in the central and western regions than eastern regions. The effect is also more prominent in non-first-tier cities than first-tier cities. The results are robust to placebo test, the Propensity Score Matching Difference-in-Difference test and the test for alternative measure of urban economic resilience. The findings show that the low-carbon pilot policy is consistent with the goal of improving urban economic resilience and technology innovation is the essential pillar of sustainable development.

## Introduction

Industrialization and urbanization would bury themselves if we don’t put enough efforts into controlling greenhouse gas (GHG) emissions. The impacts of GHG emissions include extreme weather [1, 2], food supply disruptions from drought [3], respiratory disease from smog and air pollution [4], which drastically threaten humanity and undermine the benefits of industrialization and urbanization. Among the GHGs, carbon dioxide is the main cause of the natural hazards [5].

As the largest manufacturing hub, China contributes to a third of greenhouse gases and 27 percent of carbon emissions in the world [6], among which 70 percent come from urban areas [7]. This country has just experienced industrialization and urbanization in the past three decades [8] and the impact is imminent, carbon emissions in China increased along with the economic achievements [9]. To counterbalance the bad side, take the responsibility for the world and preserve the habitability of its urban areas, China resolved to take appropriate measures to cut carbon emissions in a steady pace. At Copenhagen Conference 2009, China made its commitments to lower its carbon intensity by 40%-45% by the year 2020. After the announcement, various decarbonization initiatives have been launched [10]. The low-carbon pilot city policy is the most prominent one in city level. In 2010, 2012 and 2017, the National Development and Reform Commission announced three batches of low-carbon pilot cities, covering a wide spectrum of cities in China [11] and unfolding a brand-new episode of the carbon neutrality transition.

Although rigorous decarbonization movements have been undergoing in China, concerns about their economic impacts never vanished [12]. Fundamentally, the low-carbon pilot city policy is a shock on the economy and such a shock may lead the economic equilibrium astray from its original path. And resilience is the notion that describes the relationship between a shock and an equilibrium, how an equilibrium responds to it and what is the relevant aftermath. It has three implied meanings, which are avoiding the shock altogether, withstanding the shock when it comes and dampen the shock afterwards [13]. By and large, the country is highly reliant on fossil fuel energy to facilitate electricity supply and changes in electricity system triggered by the low-carbon pilot policy will introduce an immediate shock to the energy supply and thereby the Chinese economy [14, 15]. And it might also strike the stability of the economy and hurt the economy’s capacity to withstand shocks, which is represented by urban economic resilience. In extant literature, a range of shocks on the economy have been investigated, such as the pandemic [16], competitions from globalization [17] and industrial transformations [18]. However, the shock of the low-carbon pilot policy on the economy has not been investigated so far.

Many scholars and policy makers may still wonder, as far as the low-carbon pilot policy is concerned, will there be additional implicit cost on the stability of the economy? Specifically, will we lose the capacity to withstand external shocks after the low-carbon pilot policy has been introduced? After all, the first intention of low-carbon initiative is to improve sustainability and promote correspondence between individuals’ well-being and environmental protection. Since the stability of the economy is important to individual’s well-being, we ought to ensure that we will not sacrifice it for the environment and avoid putting the cart before the horse. To answer these questions, the author scrutinizes the impact of the low-carbon pilot policy on urban economic resilience and investigates the underlying mechanism.

The novelty of this paper is threefold. Firstly, most researches on low-carbon pilot policy mostly focus on the design [19–21], development [22–24] and environmental effects [25–28]. There is not much effort put into the relationship between low-carbon pilot policy and the urban economic resilience. This paper fills the gap in the literature between low-carbon pilot policy and urban economic stability.

Secondly, although there are many papers focused on the effects of various shocks on the economy equilibrium, which is where the notion of resilience resides, few have concentrated on the impact of shocks on the resilience itself. As the economic resilience is the capacity to resist, contain, and dampen shocks, it can also be changed by the shock, which is to say, an economy’s capacity of dealing with shocks can also be changed by the shock. The investigation on the relationship between low-carbon pilot policy and the urban economic resilience is a specific example of the idea that the an economy’s capacity of dealing with shocks can be changed by the shock itself and hopefully it may evoke more academic attention in this area.

Thirdly, this paper facilitates the risk management process for policy makers on a low carbon transition. Practically the world needs evidence on the economic consequences of low carbon initiatives, whereas extant studies on this subject are still scarce. If it is bad for economic stability, we can think of ways to neutralize its effect and prepare in advance in the process of decarbonization. If it is good for it, we can promote low carbon transition with higher spontaneity. Anyways, it is good to know the policy consequences beforehand.

The remainder of the paper is structured as follows: Section 2 narrates the context of the low-carbon pilot policy and illustrates the theoretical model depicting how the low-carbon pilot policy would affect urban economic resilience. Section 3 introduces the methods and data used in empirical analysis. Section 4 shows and interprets the empirical results and section 5 investigates the underlying mechanism between low-carbon pilot policy and urban economic resilience. Section 6 presents results of the robustness tests and section 7 concludes the paper with policy implications.

## Policy context and theoretical analysis

### Policy context

Containing carbon emission is no easy task for China, given that 85% of the energy supplies are reliant on fossil fuels [11]. The low-carbon transition in China is a huge systemic project that covers both the demand side and supply side among various industries. On the demand side, the measures include upgrading energy utilization efficiency of power intensive industries, updating emission reduction techniques and escalating the development of new-energy vehicles. On the supply side, the government has to support the renewable energy industry at an early stage, including photovoltaic generation facilities and wind farms. Since there are so many things to do, it requires nationwide correspondence across various regions. And the low-carbon pilot policy is the one to ensure an orderly implementation of the national decarbonization campaign. In fact, the low-carbon pilot policy is the crucial component of China’s national development plan. In November 2010, the 12^th^ Five-Year Plan initially proposed the idea of constructing ecological civilization and the first batch of low-carbon pilot policy, which was announced by the National Development and Reform Commission of China (NDRC), was the cornerstone of the plan. The first batch only includes five provinces and eight cities. In 2012, the NDRC announced the second batch, expanding another twenty-seven cities and an additional province. In 2017, the third batch was released to cover the county areas across cities. With these preparations and experience at hand, in September 2020, China announced its ’30–60 goal’ to hit carbon emission peak by 2030 and reach carbon neutrality by 2060, unfolding the long-term campaign in combating greenhouse gases.

### Theoretical analysis from economic theory

How would the low-carbon city pilot policy impact urban economic resilience in China? To answer the question, we need to understand the characteristics of the Chinese economy thoroughly. The world has witnessed China’s economic boom after forty years of the reform and opening-up policy. In academia, economists never stop investigating the causes behind the economic achievements. Among the theories, Montinola et al. propose the most representative one, the “Chinese style federalism” theory [29]. They state that the political foundation and institutional reforms facilitate decentralization and the market thrives with local governments pursuing economic prosperity. The Chinese style federalism preserves the market, introduces competition and starts the era of investment-driven economic boom. The competition among cities stimulates local governments to attract foreign investments and promote the economy. And China’s economic development is the result of all city managers seeking maximum economic surplus. In this model, we follow the setting of city competition and regard cities as independent and homogeneous economic agents (denoted by i) competing with each other. Assume that the city manager can fully decide his optimal carbon emission quantity (denoted by q). As is often the case, carbon dioxide emission by one city does not directly influence the economic outcome of other cities. In other words, excessive carbon emission is considered to be “bad” public goods in the concept of economics, rather than negative externalities. The attribute “bad” means to impose additional costs on every agent in the game, instead of delivering benefits and welfare to each market participant. Assume that city i pays real cost c(q) for q amount of carbon dioxide emission and correspondingly induces an amount g(q) of the implicit loss, we can easily set up the optimization problem as:

$$Max\sum_{i}g_{i}(q)-c(q)$$ (1)

From the first order condition, we can get the optimal solution:

$$\sum_{i}g_{i}^{'}(q^{*}){=c}^{'}(q^{*})$$ (2)

**Fig 1:**
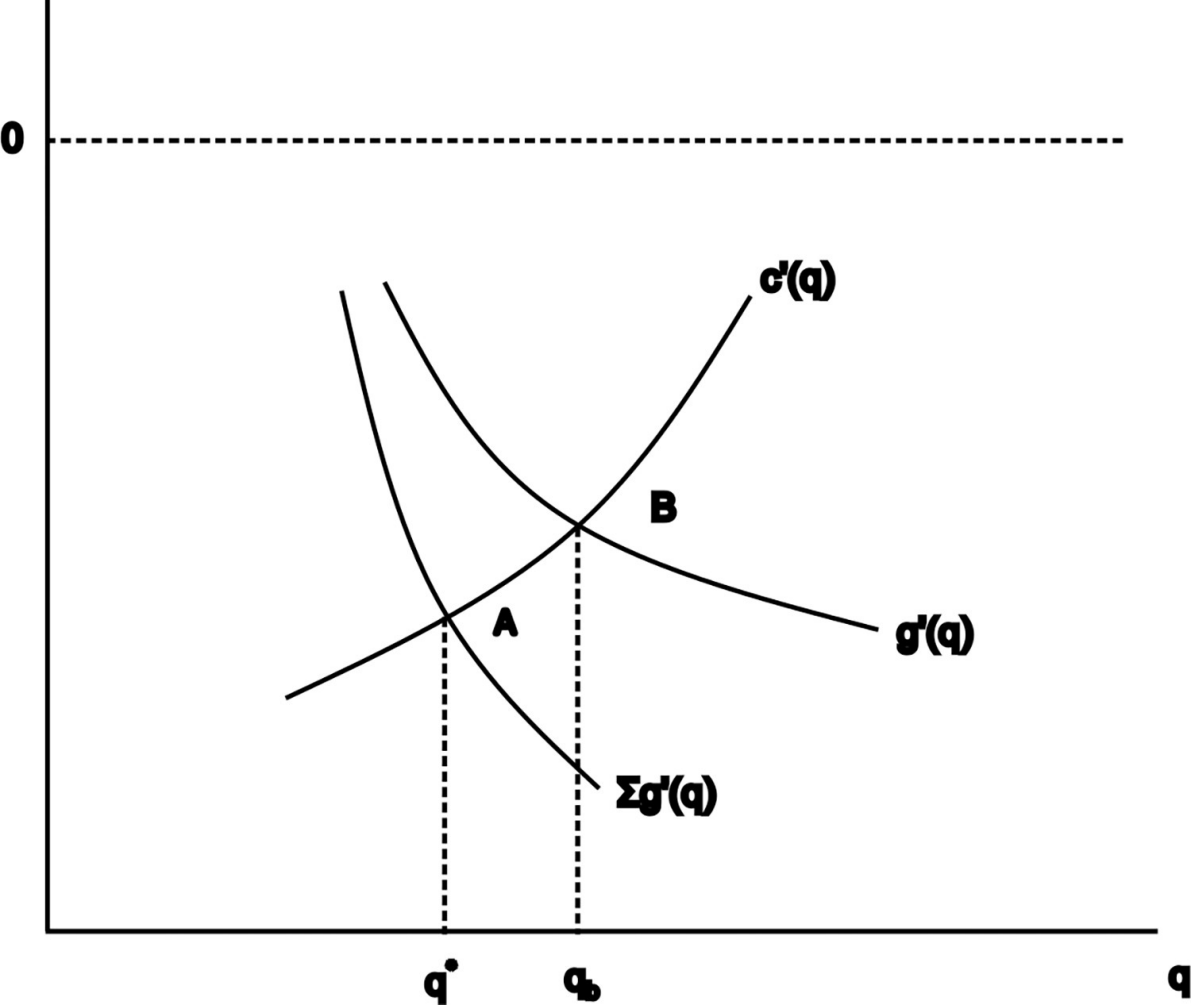
Theoretical analysis of carbon dioxide emission by homogeneous cities.

That is, the optimal amount of carbon dioxide emission is equivalent to the marginal implicit cost of each city. However, the actual amount of carbon dioxide emission will always exceed the optimal amount. As is portrayed in Fig 1, curve c’(q) indicates the marginal cost of city carbon dioxide emission and curve g’(q) denotes individual city’s implicit loss for additional amount of carbon dioxide emission. The curve Σg’(q) represents sum of all cities’ implicit loss for additional amount of carbon dioxide emission. Contrary to the standard public goods, the “bad” public goods involve negative marginal utility g’(q) rather than positive one, thereby exhibiting a lower aggregate emission curve Σg’(q) than individual emission curve g’(q). The intersection of curve c’(q) and curve Σg’(q) which is portrayed as point A is the optimal amount of carbon dioxide for best social welfare. And the intersection of curve c’(q) and curve g’(q) which is portrayed as point B is the optimal amount of carbon dioxide for every individual and homogeneous city. As we can see from Fig 1, the optimal carbon dioxide emission for individual city q_b_ is always larger than the optimal social welfare one q*. That is, the total amount of carbon dioxide emission by individual city managers’ decisions will deviate from the social welfare maximum amount to an inefficient level.

Theoretically, if cities emit carbon dioxide according to the quota of optimal quantity, it should do no harm to the society or the environment and contribute little to global warming issue. The problem is the excessive emission, which is expressed as the difference between q_b_ and q*. To make the economy more resilient and ecologically friendly, the government has to launch public policy to address the oversupply of carbon dioxide emission by quantitative intervention. Considering that the granted low-carbon pilot cities are increasing in numbers, the three batches of carbon pilot policies which are issued by the National Development and Reform Commission of China serve as the quantitative approach in the intervention of carbon dioxide emission. Based on this, this paper proposes the Hypothesis 1:

Hypothesis 1: Low-carbon pilot policy can help improve the urban economic resilience.

### Mechanisms of the influence

According to the analysis above, it is straightforward that the partial equilibrium of individual city i is mutually determined by the marginal cost c’(q) and the implicit loss g’(q), which is induced by carbon emissions. As the marginal cost c’(q) is affected by the emission reduction technology, technology updates on emission reduction will influence the function parameter of c(q), thereby changing the local equilibrium output of carbon emission, reducing the gap between the general equilibrium and the partial equilibrium, and eventually improving the economic resilience of the city.

From a practical perspective, if a city invests more resources in the research of emission reduction and the technology update reduces energy consumption for equivalent amount of economic output, the economic output would be greater for the same amount of energy consumption. That is, the investment in emission reduction research will improve the urban economy and increase the urban economic resilience. Based on this, this paper proposes the Hypothesis 2:

Hypothesis 2: Low-carbon pilot policy improves urban economic resilience through the mechanism of technology innovation.

Literatures have shown that R&D investment conforms to the law of diminishing marginal returns. For example, Faff et al. indicate that the R&D investment is diminishing in marginal returns regarding manufacturing industries [30]. As a sub-division of the R&D, the investment in technology of carbon emission reduction is also marginal diminishing. Generally, the geographical distribution of economic activities is not balanced and eastern regions are more developed than central and western regions in China. Thus, the economic gain of regional R&D investment for western region in China is much higher than that of eastern regions [31]. Consequently, the decline of the c(q) level in eastern regions is not as significant and effective as that in the central and western regions. Similar difference appears in the effect of low-carbon pilot policy on urban economic resilience between first-tier cities and non-first-tier cities. Therefore, the stimulating effect of low-carbon technology development in underdeveloped areas such as central and western regions and non-first-tier cities is greater than that of cities in eastern regions and first-tier cities. Based on this, this paper proposes Hypothesis 3:

Hypothesis 3: Low-carbon pilot policy is more effective on improving the urban economic resilience of cities in central and western regions and non-first-tier cities, than that of cities in eastern regions and first-tier cities.

## Methodology and data

### Data

The dependent variable of the research is urban economic resilience. A lot of researchers have contributed to the conceptualization of urban economic resilience [32]. Although the terminologies may vary, they mostly fall into three kinds of interpretations: (1) the ability to bounce back from shocks and disruptions, (2) the ability to absorb shocks and (3) adaptability in the anticipation of disturbances [13, 33]. The first appearance of urban economic resilience in empirical examinations comes from Tan et al. [34], where he uses a composite index of persistence, adaptability and transformation as the economic resilience indicator. Each dimension corresponds to an aspect of conceptualization of the economic resilience. Persistence is the ability to stay robust and unchanged when confronting a shock and it is also the economic resilience beforehand or a pre-shock resilience. Adaptability is the capacity to absorb the shock and restore resumption of the growth path. Transformation is of the re-orientation dimension and is associated with a structural reform that will lead to a satisfactory transition in the long run. Following Tan et al. [34] and Li and Huang [35], this paper concretizes these three dimensions and constitutes a similar indicator in assessing and evaluating urban economic resilience. The calculation methods are shown in Table 1. The weights for each of the coefficients mostly come from Yu et al. [36].

**Table 1:** Components and coefficients of urban economic resilience.

Category	Index	Calculation	Correlation	Weight
Persistence	Economic scale	Regional GDP per capita	positive	0.1216
	Residents’ risk tolerance	Disposable income per capita	positive	0.0598
	Industrial structure	The proportion of employees in the secondary sector	negative	0.0857
	Foreign investment dependence	Foreign investment/ regional GDP	negative	0.0121
	Environmental sustainability	Green coverage in urban area	positive	0.0376
Adaptability	Economic growth	Regional GDP growth rate	positive	0.0720
	Financial soundness	General budget revenue/expenditure of local finance	positive	0.0723
	Robustness of financial institutions	End-of-year balance of deposits/balance of loans in financial institutions	negative	0.0679
	Fixed asset investment	Logarithm of fixed asset investment	positive	0.0284
	Domestic demand	Total retail sales of consumer goods/regional GDP	positive	0.0214
Transformation	Urbanization	Constructed area/administrative jurisdiction area	positive	0.0035
	High-end industry Index	Proportion of 1st industry×1+proportion of 2nd industry ×2+proportion of 3rd industry ×3	positive	0.1176
	Academic scholar	Number of researchers/Total employees	positive	0.0813
	Human capital adequacy	Number of enrolled colleges and universities students/total population	positive	0.1524
	Industrialization of academic research	Expenditure on science, technology and innovation/Regional GDP	positive	0.0145
	Innovation	Number of granted patents per capita	positive	0.0519

The explanatory variable of this paper is the low-carbon pilot policy. The carrying out of the low-carbon pilot policy is going through three batches. In 2010, the National Development and Reform Commission (NDRC) announced the Notice on Carrying Out Low-carbon Province and City Pilot (No. 1587 [2010]). In 2012, the NDRC released the Notice on Carrying Out the Second Group of Low-carbon Province and City Pilot (No. 3760 [2012]). In 2017, the NDRC released the Notice on Carrying Out the third Group of Low-carbon City Pilot (No. 66 [2017]). These 3 documents mark the three batches of low-carbon pilot policy.

The first two batches of the low-carbon pilot policy are mostly around cities and provinces. The first batch of low-carbon pilot policy is issued in 2010 and entails five provinces and eight volunteer cities. The second batch of the low-carbon pilot policy is issued in 2012 and 27 cities and a province followed. The third batch of the low-carbon pilot policy is issued in 2017 and it is expanded to county level. The third batch of the pilot policy is fundamentally different from the first two in four aspects. Firstly, it is focused on county level instead of city level or province level. Secondly, it proposes the peak year for carbon emission instead of the beginning year in the first two batches. Thirdly, the peak years are distinct for the candidates rather than the same for the first two batches. Lastly, the peak years are mostly beyond 2022 which are not within the period of this empirical study, whereas few are within the period of 2004 to 2020. In order to incorporate the third batch properly, the author calculates urban economic resilience with two calculation methods: variable carbonpost only considers the first batch and second batch of the low-carbon pilot policy, and variable carbonftpost considers all three batches and takes the peak year as the policy year in the third batch. The variable carbonpost or carbonftpost is set to 1 if city i is the pilot city after the policy implementation year, and set to 0 otherwise.

In order to control other factors, this paper follows the previous papers [7, 36–39] and introduces the total population, urban industrial structure, urban medical standard, average salary level, and urban informatization level as control variables. Among them, the urban industrial structure is measured by the gross product ratio of the secondary sector to the third sector. The medical standard is measured by the logarithm of the number of doctors in the city. The level of urban informatization is described by the logarithm of the number of Internet users in the city.

After removing samples of cities with adjusted administrative areas and missing values, the final sample comprises a panel database of 277 cities over the period of 2004–2020. The data is obtained from the “China City Statistical Yearbook” and the “China Statistical Yearbook for Regional Economy”. The patent data is collected from the Chinese Research Data Services (CNRDS) database. The data from the “China City Statistical Yearbook” and the “China Statistical Yearbook for Regional Economy” is free and open to everyone. The CNRDS database is open to students and staff in universities with its subscription. All of the data is granted full permission by the user agreement of the database. This paper also follows previous research [40] and adds city and time level dummy variable to control these factors’ impact on urban economic resilience.

The descriptive analysis for all variables used in regression model is shown in Table 2. From the table, we can see that the urban economic resilience varies widely with a standard deviation of 66.11, which demonstrates the nationwide imbalance of economic development level. Other variables remain relatively stable.

**Table 2:** Descriptive statistics of the variables.

Variables	N	Median	Mean	Std	Min	Max
carbonpost	4709	0.000	0.186	0.389	0.000	1.000
carbonftpost	4709	0.000	0.186	0.389	0.000	1.000
population	4709	3.766	4.446	3.134	0.297	34.280
resilience	4709	76.430	93.000	66.110	14.150	339.100
lndoctornum	4709	4.170	4.215	0.754	1.971	7.078
indus_struct	4709	1.226	1.330	0.709	0.187	10.600
lnavewage	4709	5.981	5.896	0.607	3.904	7.523
lncyberusr	4703	12.900	12.860	1.235	5.466	17.760

### Empirical specification for the baseline model

In this paper, the author uses the difference-in-difference approach (DID) to evaluate the relationship between low-carbon pilot policy and urban economic resilience. The DID method divides the sample into 2 different categories: the treatment group and the control group, and splits the period of time into pre-intervention period and post-intervention period. The policy effect is thus extracted and evaluated from the treatment group to control group difference between pre-intervention period and post-intervention period. The DID method is effective in policy assessment since its first finding. In order to investigate the impact of low-carbon pilot policy on urban economic resilience, this paper uses urban economic resilience as the dependent variable and low-carbon pilot policy as the independent variable to construct the following benchmark regression model:

Resilienceit=α0+α1Carboni×Posti,t+α2Controli,t+γi+δt+εi,t (3)

where Resilience_it_ stands for urban economic resilience for city i and year t, and Carbon_i_ denotes the low-carbon pilot policy for city i. Carbon_i_×Post_it_ is set to 1 if city i is the pilot city after year t and set to 0 otherwise. Control_it_ is all of the control variables and γ_i_, δ_t_ is the city fixed effect and time fixed effect respectively. ε_i,t_ is the random error term that conforms to normal distribution.

### Empirical specification for the Heckman two stage model

The urban economic condition may influence the willingness to apply for the low-carbon city policy. For example, if a city is in its economic prosperity, it will be more likely to apply for the low-carbon pilot. On the contrary, if a city’s economy condition is not optimistic, it may not apply for the pilot in the first place. To control for the sample selection bias, this paper uses the Heckman two stage model and compare the results with the baseline model [41]. Specifically, this paper uses political connection as the exclusive restriction variable and takes the origin place of members of standing committee of the National People’s Congress as the proxy variable for political connection. If one member of standing committee of the National People’s Congress comes from a specific province, it is set to 1 in that province. There are 25 members in standing committee of the National People’s Congress in China. The member of standing committee of the National People’s Congress is granted with super power in China [42] and it will naturally influence the willingness of any kind of pilot policy including the low-carbon city pilot, as research shows the existence of hometown favoritism in China’s political system [43]. So the variable of political connection is associated with the willingness to apply for the low-carbon pilot, whereas it is not related to economic factors, including the urban economic resilience. With the help of the political connection variable, this paper calculates the inverse Mills ratio and utilize it to correct the bias for low-carbon city pilot in the Heckman two stage model. Then we can compare the results with the results of the baseline model.

The model specification of the first stage of the Heckman two stage model is:

$$\mathrm{Pr}(Carbon_{i}=1)=\alpha_{0}+\alpha_{1}PoliticalConn_{i,t}+\alpha_{2}Control_{i,t}+\gamma_{i}+\delta_{t}+\varepsilon_{i,t}$$ (4)

where PoliticalConn_i,t_ is the political connection variable. The second stage model is:

Resilienceit=α0+α1Carboni×Posti,t+α2imr+α3Controli,t+γi+δt+εi,t (5)

where imr stands for the variable of the inverse Mills ratio.

## Empirical results and analysis

### Parallel trend test

Although the DID approach is an effective approach in policy appraisal, it has strict restrictions that the trend of the dependent variable between the treatment group and control group in the pre-intervention period has to be parallel (also known as the parallel trend assumption). In this section, the author adds time dummy variable in the baseline model to test if it satisfies the parallel trend assumption. The model is illustrated as:

Resilienceit=α0+α1∑Pren+α2Current+α3∑Postn+α4Control+γi+δt+εi,t (6)

where the variables Pre_3_ to Pre_1_ are the time dummy variables before the launch of the first low-carbon pilot policy. The variable Current is the time dummy variable when the first low-carbon pilot policy was announced, and variables Post_1_ to Post_3_ are the time dummy variables after implementation of the first low-carbon pilot policy. Note that Current_i,t_ is the time dummy variable of the first batch of the low-carbon pilot policy, and Post_2_ is the time dummy variable of the second batch of the low-carbon pilot policy.

**Fig 2:**
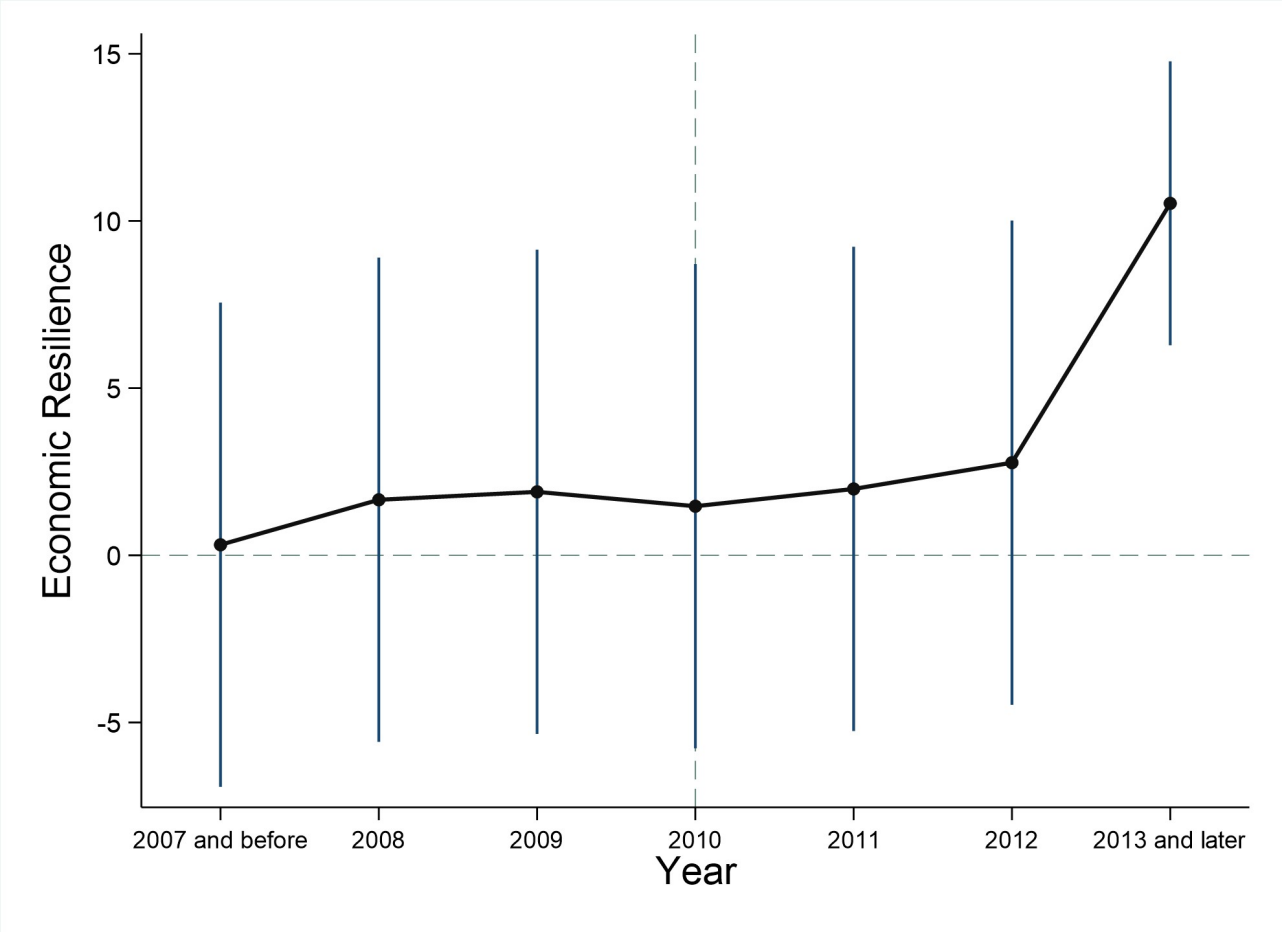
Parallel trend test.

The estimated coefficient of urban economic resilience is plotted in Fig 2. The vertical blue line is the coefficient range of 95% confidence interval. It is apparent that there is no significant difference between the low-carbon policy between treatment group and the control group before the launch of the low-carbon pilot policy. After the launch of the second batch of the low-carbon pilot policy, the coefficient of the Post_3_ dummy variable is significantly positive, indicating that the low-carbon pilot policy has played a significant role when the NDRC promulgated the second batch of pilot policy. The difference of dependent variables between the treatment group and the control group is close to zero before the first batch of pilot policy and it is significantly non-zero after year 2012, the launch of the second batch of the pilot policy. The results show that parallel trend assumption which is the prerequisite for the DID regression has been satisfied.

### Results of baseline regression

This paper uses the DID model to examine the relationship between low-carbon pilot policy and urban economic resilience. The empirical results are presented in Table 3. The first and second column illustrate regression results with carbonpost as the dependent variable, which means urban economic resilience is measured by the first batch and second batch of the low-carbon pilot cities. The first column is the empirical results without considering the control variables, and the second column is the regression results when the control variables are included in the empirical model. The third and fourth column illustrate regression results with carbonftpost as the dependent variable, which means urban economic resilience is measured by the first, the second batch of low-carbon pilot cities and the third batch of pilot cities with peak year before 2021. The third column shows the result of regression with only the main variable, while the fourth column displays the result of regression with both the main variable and the control variable. It can be seen from the table that the coefficients of carbonpost and carbonftpost are both significant and positive, indicating that the low-carbon pilot policy has promoted the improvement of urban economic resilience, thereby validating the Hypothesis 1.

**Table 3:** Result of baseline regression.

	(1) Resilience	(2) Resilience	(3) Resilience	(4) Resilience
carbon×post	7.927***	5.628***		
	(5.98)	(4.30)		
carbonft×post			7.633***	5.323***
			(5.74)	(4.06)
population		9.398***		9.390***
		(7.41)		(7.04)
lndoctornum		8.356***		8.360***
		(4.69)		(4.69)
indus_struct		-0.264		-0.248
		(-0.30)		(-0.28)
lnavewage		-14.909***		-14.972***
		(-4.88)		(-4.90)
lncyberusr		-8.949***		-8.979***
		(-10.15)		(-10.18)
_cons	91.520***	218.302***	91.580***	219.120***
	(228.39)	(10.15)	(228.51)	(10.19)
City-Fixed Effect	YES	YES	YES	YES
Time-Fixed Effect	YES	YES	YES	YES
Observations	4709	4703	4709	4703
adj. R^2^	0.893	0.899	0.893	0.898
F value	35.720	48.786	32.900	48.423

Note: (1)***,** and * mark significance at the level of 1%, 5% and 10%,respectively; (2) Values in parenthesis are the t-values of the variables.

### Heckman two stage model

The results of the Heckman two stage model are shown in Table 4. As we can see in the table, the influence of low-carbon city on urban economic resilience is still significantly positive, which is consistent with the results of the baseline model. The inverse Mills ratio is significant in column 2, which means it controls the underlying bias effectively. The values of variance inflation factor (VIFs) are below 30, indicating that no multicollinearity remains in the process [44].

**Table 4:** Results of the Heckman two stage model.

	(1) Carbon	(2) Resilience	(3) Carbon	(4) Resilience
carbon×post		11.402**		
		(2.07)		
carbonft×post				13.251**
				(2.50)
politicalconnc	-0.239		-0.246	
	(0.48)		(-0.51)	
population	-0.021	-12.136***	-0.019	-12.056***
	(0.04)	(-5.93)	(-0.46)	(-5.90)
lndoctornum	0.261	47.405***	0.249	46.609***
	(0.17)	(5.96)	(1.42)	(5.87)
indus_struct	-0.001	0.003**	-0.001	0.003**
	(0.01)	(2.36)	(-0.22)	(2.35)
lnavewage	-0.001	0.287***	-.001	0.288***
	(0.01)	(7.87)	(-0.06)	(7.86)
imr		20.298*		16.607
		(1.81)		(1.47)
_cons	-1.325**	-160.252***	-1.285**	222.489***
	(0.57)	(-5.33)	(-2.27)	(8.79)
Fixed Effect	YES	YES	YES	YES
VIFs		1.89		1.78
adj. R^2^		0.801		0.799

Note: T-value in parenthesis in column (2) and (4). Z-value in parenthesis in column (1) and (3). Use robust standard errors clustered to province and year.

## Transmission mechanism and heterogeneity test

### Transmission mechanism

The previous section has concluded that low-carbon pilot policies have a positive impact on urban economic resilience. However, it is still not clear through which channel will the low-carbon pilot policies influence urban economic resilience. In order to gain an in-depth understanding of the role of low-carbon pilot policies’ effect on urban economic resilience, this section examines the transmission process of low-carbon pilot policies on urban economic resilience especially through urban innovation activities. Existing literatures have proved that the urban economic resilience grows with the intensity of urban innovation activities. For example, Trembaczowski suggests that knowledge and ability of creation are preconditions of regional economic development and competitiveness. Knowledge stimulates urban innovation and makes the city a learning region [45]. Sedita et al. find that knowledge-based economic activities contributes to resilience [46]. Moreover, the fact that higher intensity of urban innovation activities leads to better urban economic resilience is also in line with the common sense in the economic theory. Therefore, the first half of the logic chain is already given and this paper only needs to prove the second half. By demonstrating the impact of low-carbon pilot policies on urban innovation activities, this paper can verify that whether low-carbon policies enhance urban economic resilience through the channel of stimulating urban technology innovation.

This paper takes the number of a city’s granted invention patent and granted utility models as the measure of city’s innovation level. There are 3 kinds of patents in China, which are the invention patent, utility models and industrial designs. As the industrial designs do not stand for true innovation, I pick the first two, which are the invention patent and utility models to stand for technology updates. The measurements of the invention patent and utility model are their granted numbers by the authority each year. The regression results are shown in Table 5. The dependent variable invention denotes a city’s granted invention patent and dependent variable utility represents a city’s granted utility models. Column 1 and 3 are results with only low-carbon pilot policy as the main independent variable and column 2 and 4 are results with both low-carbon pilot policy and control variables. From Table 5, the impact of low-carbon pilot policies on the urban innovation level is positive and significant, no matter which proxy variable the urban innovation level is measured by (the number of a city’s granted invention patent or a city’s granted utility models). The result verifies the Hypothesis 2 proposed in Section 2.

**Table 5:** Results of the mechanism analysis.

	(1) Invention	(2) Invention	(3) Utility	(4) Utility
carbon×post	10.728***	8.220***	30.058***	22.302***
	(11.25)	(9.61)	(10.81)	(9.32)
population		23.455***		79.542***
		(28.29)		(34.30)
lndoctornum		0.174		5.994*
		(0.15)		(1.84)
indus_struct		1.702***		5.116***
		(2.98)		(3.20)
lnavewage		-7.311***		-31.682***
		(-3.66)		(-5.66)
lncyberusr		-9.053***		-24.949***
		(-15.70)		(-15.47)
_cons	3.825***	56.545***	17.933***	141.272***
	(13.28)	(4.02)	(21.35)	(3.59)
City-Fixed Effect	YES	YES	YES	YES
Time-Fixed Effect	YES	YES	YES	YES
Observations	4709	4703	4709	4703
adj. R^2^	0.611	0.696	0.553	0.679
F value	126.613	231.919	116.892	312.782

Note: (1)***,** and * mark significance at the level of 1%, 5% and 10%,respectively; (2) Values in parenthesis are the t-values of the variables.

### Heterogeneity test

The endowment condition and geographical feature of cities vary widely in China. Firstly, there are vast landform differences across the Heihe-Tengchong Line. The Heihe-Tengchong Line (also known as the Hu’s line) is a line on topography dividing China into two completely distinct terrains. The landform on northern side of the Hu’s line is mostly plateau, desert or valleys and the southern side is mostly plain, which is more suitable for living and economic activity. In addition, the phenomenon of city tier hierarchy is especially prominent in China. The first-tier cities, which are the four renowned mega-metropolitans in China, (Shanghai, Guangzhou, Shenzhen) mostly locate near seaports and benefit from the dividends of sea trading. The only exception of non-port first-tier city, Beijing, is the capital city and political center of the nation. And many second-tier cities locate near the Yellow River or the Yangtze River, for example, big cities such as Chongqing, Wuhan, Hangzhou locate in the Yangtze River basin, and cities such as Xining, Lanzhou, Yinchuan, Baotou, Zhengzhou, Jinan are in the Yellow River basin. Therefore, it is intuitive to think, will the city tier hierarchy influence urban economic resilience? The following section explores the relationship between low-carbon pilot policy and urban economic resilience in geographical positions such as central, western region and eastern region, first-tier cities and non-first-tier cities. The results are shown in Table 6. From the table, the regression results show that the low-carbon pilot policy has significantly improved the economic resilience of cities in the central and western regions, while the impact on the economic resilience level of cities in the eastern region is insignificant. At the same time, the low-carbon pilot policy has significantly improved the urban economic resilience of non-first-tier cities, while the impact on first-tier cities is not significant. The results validate that the stimulating effect of low-carbon pilot policy on urban economic resilience is mainly achieved by technology innovation and the technology in the eastern region and first-tier cities is already fully prepared, leaving little room for improvements in these regions. Therefore, the technology channel does not work for the eastern region and first-tier cities and the coefficients are insignificant in these cases. The empirical results in the heterogeneity test support the Hypothesis 3.

**Table 6:** Heterogeneity test.

	By Geographical Positions		By City Tier Hierarchy	
Resilience	Central and west regions	East region	First-tier cities	Non-first-tier cities
carbon×post	7.856***	-2.084	7.685	3.342**
	(5.11)	(-0.64)	(0.18)	(2.34)
population	9.079***	1.981	-22.532	6.146***
	(6.50)	(0.53)	(-1.03)	(4.16)
lndoctornum	7.355***	-11.820*	117.228	9.299***
	(3.98)	(-1.89)	(1.50)	(4.85)
indus_struct	-1.429	-0.663	-16.183	-1.441
	(-1.62)	(-0.18)	(-0.28)	(-1.54)
lnavewage	3.203	-76.172***	44.398	-15.433***
	(1.02)	(-7.14)	(0.41)	(-4.69)
lncyberusr	-5.246***	-9.626***	0.819	-7.050***
	(-5.02)	(-4.45)	(0.05)	(-7.22)
_cons	60.873***	756.312***	-483.265	208.515***
	(2.69)	(10.83)	(-0.56)	(9.01)
City-Fixed Effect	YES	YES	YES	YES
Time-Fixed Effect	YES	YES	YES	YES
Observations	3225	1478	102	4601
adj. R^2^	0.880	0.862	0.723	0.880
F value	19.950	16.345	0.483	25.153

Note: (1)***,** and * mark significance at the level of 1%, 5% and 10%,respectively; (2) Values in parenthesis are the t-values of the variables.

## Robustness test

### Placebo test

As the empirical tests in Section 4 suggest, the low-carbon pilot policy improves urban economic resilience. One may ask, does the low-carbon pilot policy distinctly improve urban economic resilience? Or any kind of disturbance will cause the growth of urban economic resilience? The placebo test will address the issue by generating random policy cities and random policy years. Regressions with the same model specification will be tested again except that randomly generated low-carbon pilot policy dummy pairs of city and year are used. If the regressions of placebo test remain positive and significant, it means the causality is from random factors and does not genuinely exist. If the coefficient of the regression distributes normally around the value of zero, it means random factors do not improve urban economic resilience, which indicates the validation of the empirical results of the baseline model in Section 4. Thus, in order to minimize the impact of unobservable factors on the regression results, in this section, the author will analyze the robustness of the results with placebo tests.

**Fig 3:**
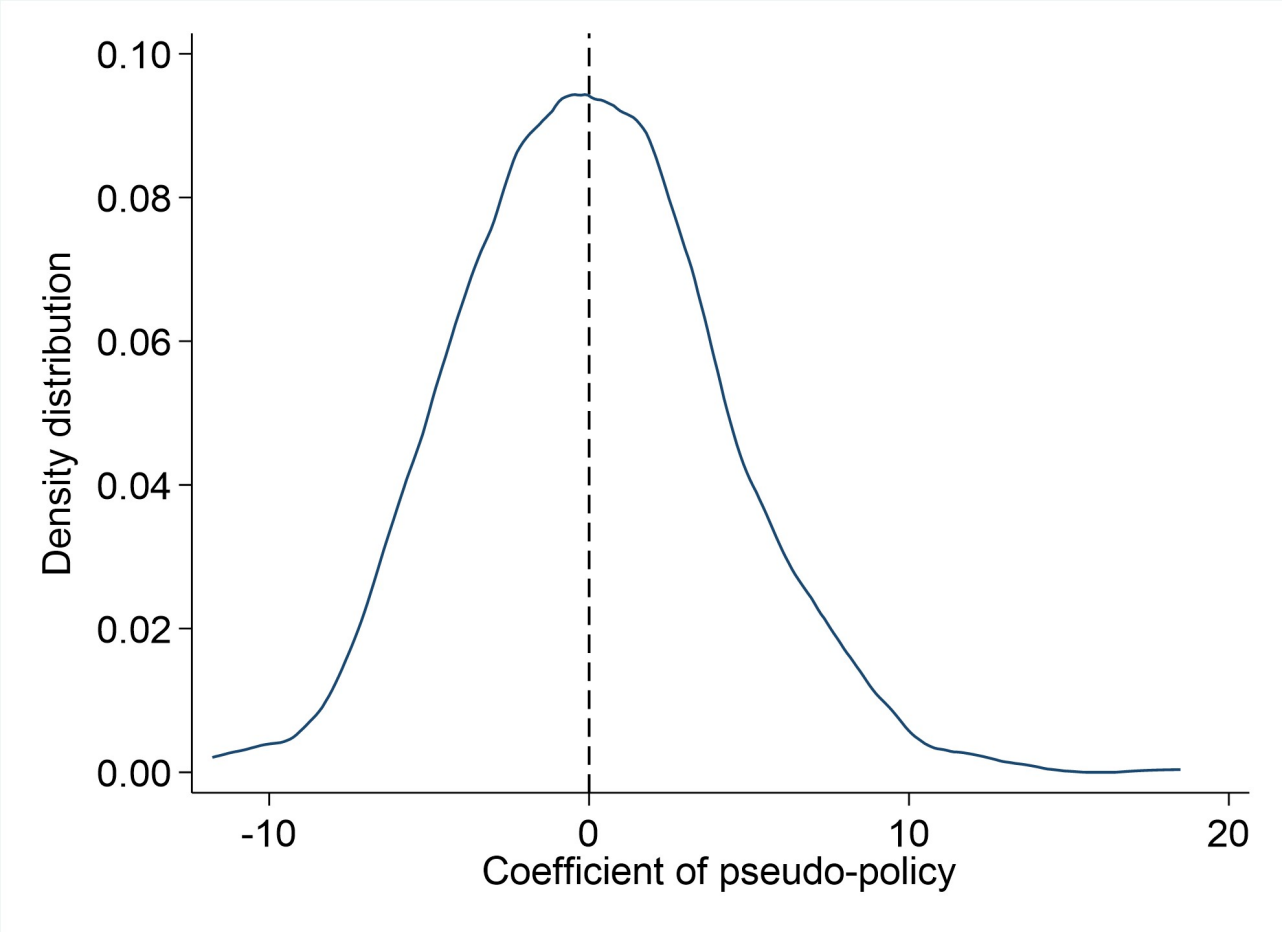
Density distribution of coefficient of pseudo-policy.

**Fig 4:**
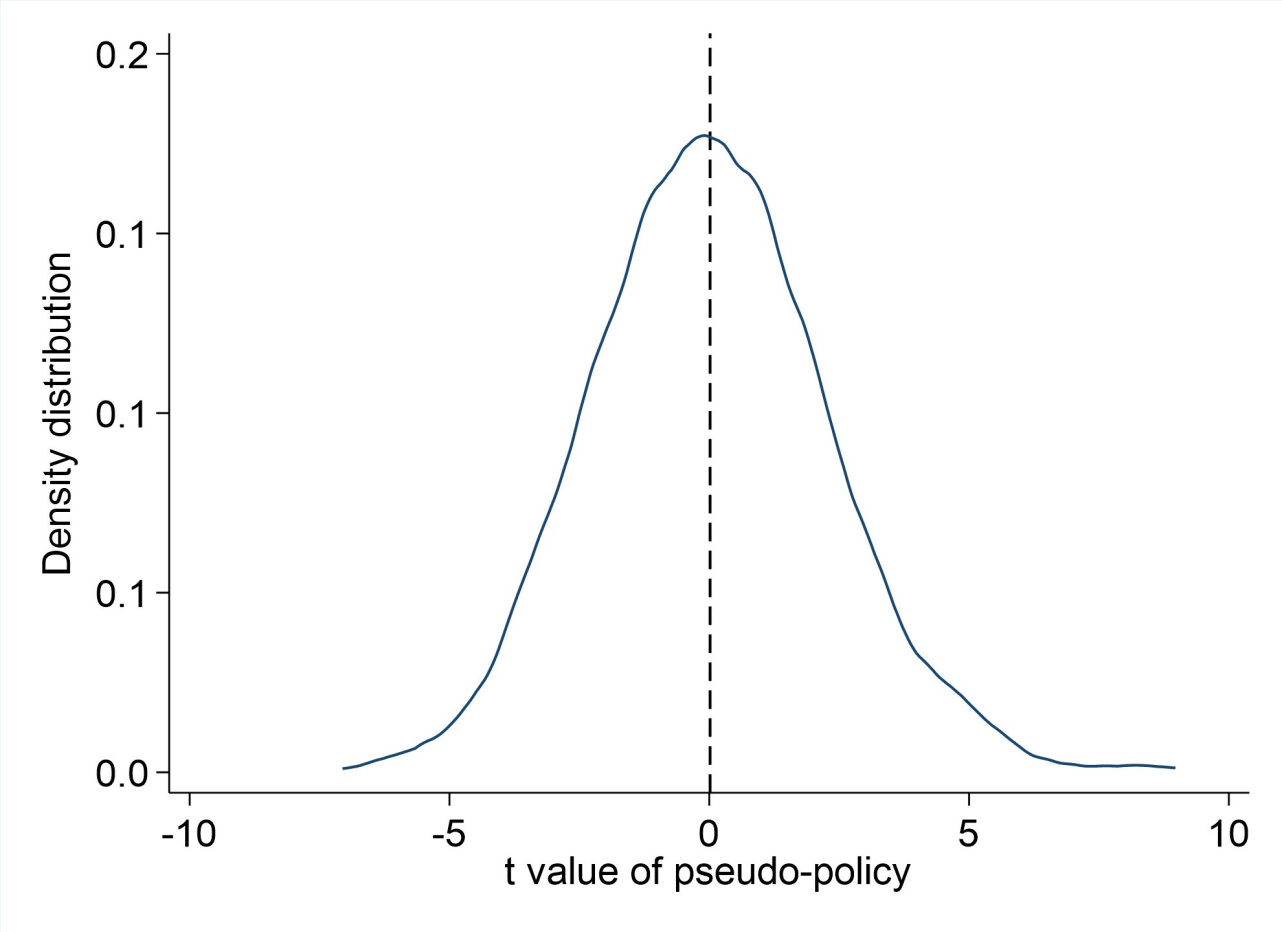
Density distribution of t value of pseudo-policy.

The placebo tests start with generating pairs of pseudo low-carbon pilot city and implementation year. There are 69 and 23 pilot cities for the first two batches of the genuine low-carbon pilot policy. The third batch is ignored as most peak years of cities in the third batch are beyond 2022. To be consistent with the baseline specification and make the result of the placebo test more comparable, this paper generates 2 pseudo policy years with 69 pilot cities and 23 pilot cities for each respective pseudo policy implementation year. In order to prevent the impacts of extreme results, the author reiterates the placebo tests for 1000 times. This paper presents the density graph of the coefficient of the variable carbonpost on Fig 3 and the density graph of the t value of the variable carbonpost on Fig 4. It shows that the density graph for pseudo low-carbon pilot city coefficient distribution and t value distribution follow a normal distribution around zero which means the artificially generated pseudo policy will not improve the urban economic resilience. The results show that the impact of low-carbon pilot policy on urban economic resilience is not caused by unobservable factors.

### PSM-DID test

**Fig 5:**
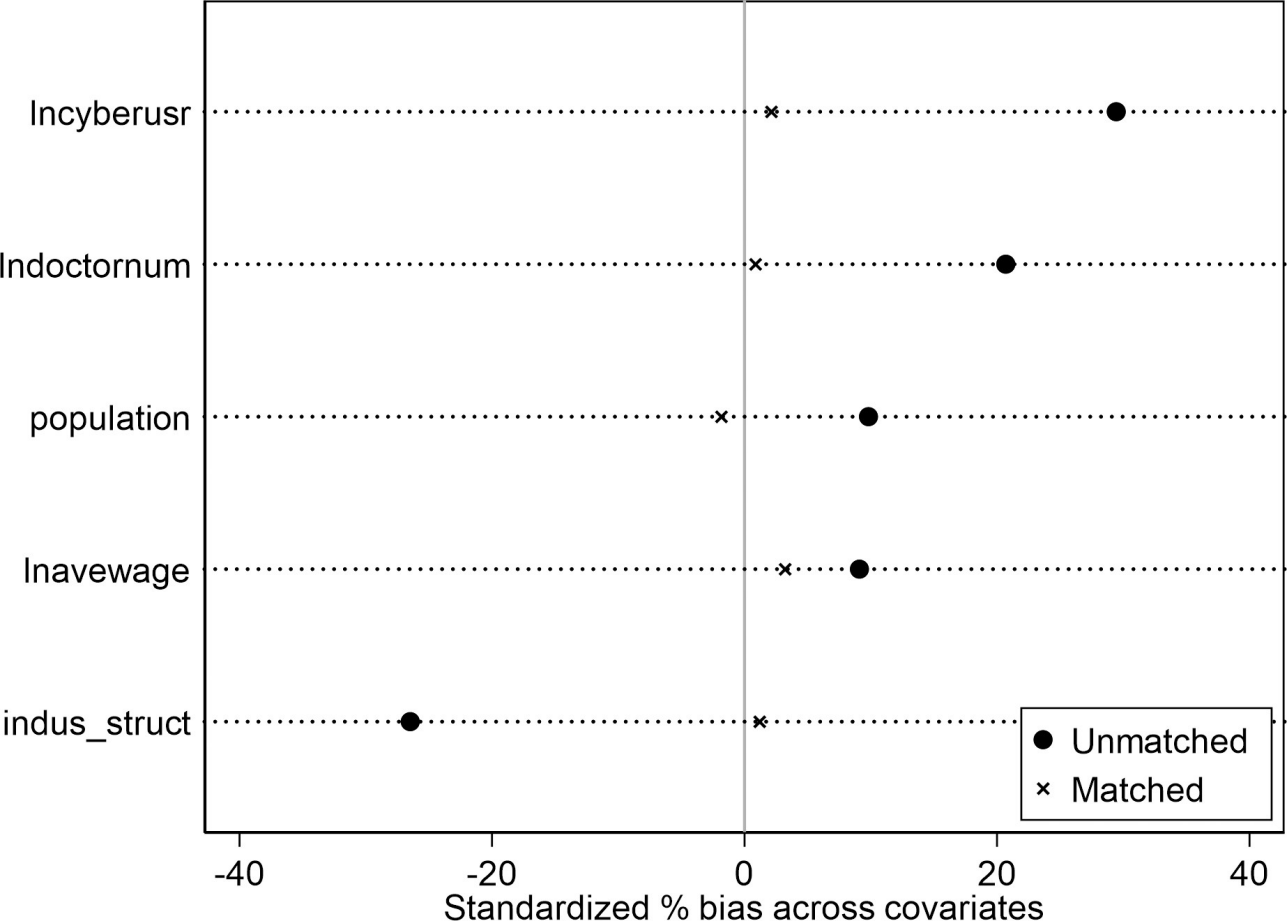
Differences before and after matching.

In order to eliminate the possible selection bias of the difference-in-difference model, this paper introduces the propensity-score-matching difference-in-difference (henceforth PSM-DID) method in order to improve the robustness of the research results. Firstly, this paper examines whether it is appropriate to apply the PSM-DID approach. To test the effect of propensity score matching, this paper carries out the balance test. The results of the balance test are shown in Table 7. The results in the table show that the standard error of each variable after matching is less than 10%, and is smaller than the standard error before matching. The standard error of each variable has been greatly reduced by a range of 64.8% to 95.8%, and all the variables do not reject the null hypothesis that “there is no systematic deviation of variable values before and after matching”. Table 7 also shows that the pseudo R^2^ of the regression results after matching is significantly smaller. These results show that propensity score matching approach works well in bringing down the standard deviation, and the DID regression can be more effective with this matched sample. Fig 5 also displays a straightforward illustration of the effectiveness of the propensity-score-matching process. The dots show the unmatched sample’s standardized biases for each of the variables and the crosses show the matched sample ones. From the figure it is easy to see that the PSM process significantly decreases the standardized bias, indicating a sample improvement for the DID regression after the PSM process.

**Table 7:** Results of the balance test.

Variable	Sample	Mean Difference		Bias Reduction		
		Treatment Group	Control Group	Bias	Reduct	t-test
population	Unmatched	4.663	4.3331	9.8	81.3	3.44***
	Matched	4.655	4.7164	-1.8		-0.52
lndoctornum	Unmatched	4.320	4.160	20.7	95.8	6.97***
	Matched	4.318	4.312	0.9		0.25
indus_struct	Unmatched	1.213	1.393	-26.5	95.5	-8.28***
	Matched	1.214	1.206	1.2		0.40
lnavewage	Unmatched	5.931	5.876	9.1	64.8	2.97***
	Matched	5.932	5.913	3.2		0.91
lncyberusr	Unmatched	13.103	12.736	29.4	92.8	9.81***
	Matched	13.104	13.077	2.1		0.61
	PSM R^2^	LR Chi^2^	P value	Mean Bias		
Unmatched	0.034	209.31***	0.000	19.1		
Matched	0.000	1.92	0.861	1.8		

**Fig 6:**
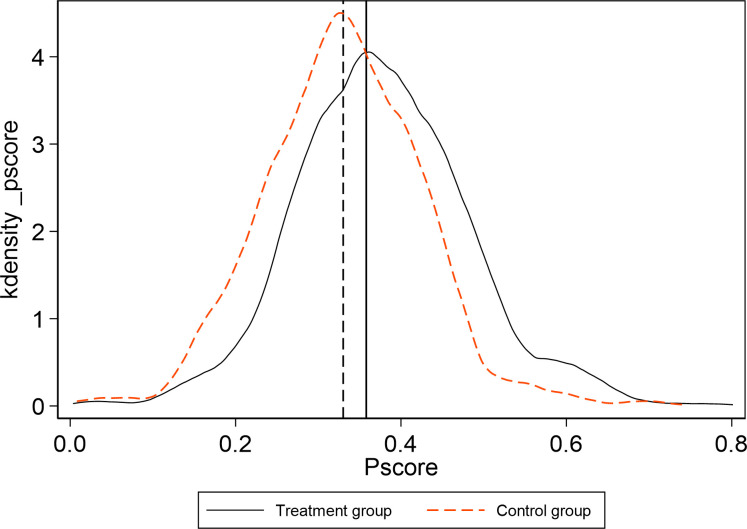
Kernel density of PSM in the treatment group and the control group before matching.

**Fig 7:**
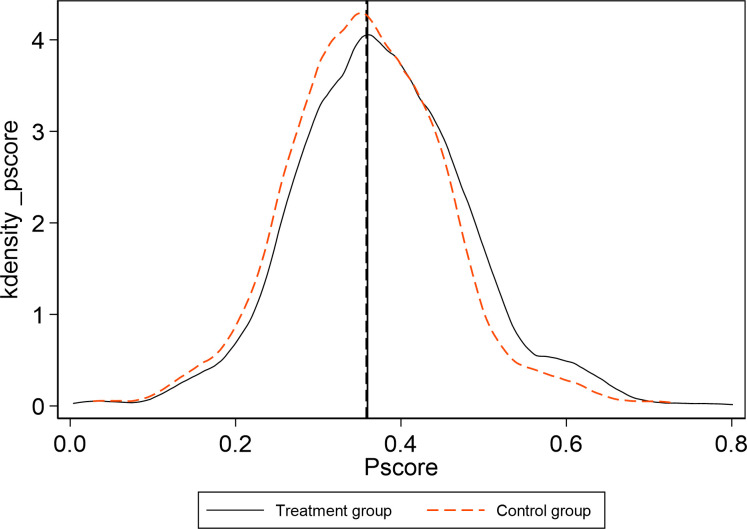
Kernel density of PSM in the treatment group and the control group after matching.

At the same time, Fig 6 shows the probability density of the unmatched sample and Fig 7 shows the probability density of the matched sample. There are apparent differences between the treatment group and the control group before matching, while density curves of the treatment group and the control group are much closer after matching, which suggests that the matched sample with propensity score matching approach will be better in the DID regression than the unmatched sample.

Table 8 presents the results of the robustness test with the PSM-DID method. The first column is the regression results of the fixed effects model, and the second to the fourth columns are the results of regression estimation using distinct types of PSM samples. The second column is the result of regression using matched samples with non-zero weights, the third column is the result of regression using matched samples that satisfy the common support hypothesis, and the fourth column is the result of regression using frequency weighting samples. It can be seen from the table that after the PSM matching, the number of observations declined compared with the benchmark regression, despite few in reduction amount. The results show that the coefficient of carbon×post using the PSM-DID approach is still positive and significant, which demonstrates the robustness of the baseline result.

**Table 8:** Results of the PSM-DID robustness tests.

	(1) FE	(2) PSM-DID1	(3) PSM-DID2	(4) PSM-DID3
carbon×post	6.710***	5.487***	6.476***	4.421***
	(4.360)	(2.633)	(4.200)	(2.789)
population	10.252***	13.185***	10.186***	12.231***
	(6.873)	(6.287)	(6.828)	(10.210)
lndoctornum	8.733***	4.340	8.882***	7.101***
	(4.162)	(1.432)	(4.231)	(3.470)
indus_struct	-1.757*	-1.811	-1.619	-2.317*
	(-1.710)	(-1.000)	(-1.573)	(-1.907)
lnavewage	-16.157***	-27.163***	-16.877***	-34.077***
	(-4.491)	(-4.138)	(-4.445)	(-8.191)
lncyberusr	-8.877***	-6.160***	-9.625***	-5.783***
	(-8.555)	(-3.932)	(-8.732)	(-5.972)
_cons	221.647***	255.440***	235.068***	284.469***
	(8.757)	(6.070)	(8.964)	(10.341)
Observations	4703	2178	4699	4364
adj. R^2^	0.871	0.875	0.871	0.891
F value	39.859	17.702	40.644	48.340

Note: (1)***,** and * mark significance at the level of 1%, 5% and 10%,respectively; (2) Values in parenthesis are the t-values of the variables.

### Test for alternative measure of urban economic resilience

Although it is more comprehensive to evaluate the urban economic resilience with all of the three dimensions (resistance, adaptability and transformation), there are still many papers only measuring urban economic resilience in terms of adaptability [47–50]. Thus, in order to prove it is independent of specific measurements and verify the robustness of the regression results again, this paper uses this alternative measure of urban economic resilience and examines the relationship again. The calculation method is as follows:

Resilience*=[(Yi,t−Yi,t−1)/Yi,t−1−(Yn,t−Yn,t−1)/Yn,t−1]/(Yn,t−Yn,t−1)/Yn,t−1 (7)

where Y_i,t_ represents the regional real GDP of city i in period t, Y_i,t-1_ denotes the regional real GDP of the previous period, Y_n,t_ stands for the real national GDP in period t and Y_n,t-1_ represents the national GDP in the previous period. This paper also detrends the variable to make necessary simplifications:

Resiliencei,t=Resilience*i,t−∑iNResilience*i,t/N (8)

The empirical results are shown in Table 9. Column 1 shows the regression of low-carbon pilot policy on the alternative proxy variable of urban economic resilience, without control variables. Column 2 shows the regression of low-carbon pilot policy on the alternative proxy variable of urban economic resilience, with control variables. The coefficient of variable carbon×post is 0.114 in column 1 and 0.141 in column 2. Column 3 shows the regression of low-carbon pilot policy with the cities of third batch on the alternative proxy variable of urban economic resilience, without control variables. Column 4 shows the regression of low-carbon pilot policy with the cities of third batch on the alternative proxy variable of urban economic resilience, with control variables. The coefficient of variable carbonft×post is 0.119 in column 3 and 0.144 in column 4. Table 9 shows that all the regression results of variable carbon×post and carbonft×post are positive and significant, which means that the low-carbon pilot policy improves urban economic resilience measured in various approaches. This result confirms the robustness of the empirical result and shows that hypothesis 1 is valid despite distinct measurements of urban economic resilience.

**Table 9:** Robustness test.

	(1) Resilience*	(2) Resilience*	(3) Resilience*	(4) Resilience*
carbon×post	0.114**	0.141***		
	(2.08)	(2.59)		
carbonft×post			0.119**	0.144***
			(2.16)	(2.64)
lnavewage		0.634***		0.634***
		(4.98)		(4.98)
lncyberusr		0.091**		0.091**
		(2.38)		(2.39)
privecon		0.001**		0.001**
		(2.20)		(2.20)
population		0.335***		0.335***
		(6.24)		(6.23)
lndoctornum		0.245***		0.244***
		(3.27)		(3.27)
_cons	-0.181***	-7.728***	-0.182***	-7.728***
	(-10.78)	(-8.46)	(-10.83)	(-8.46)
City-Fixed Effect	YES	YES	YES	YES
Time-Fixed Effect	YES	YES	YES	YES
Observations	4427	4423	4427	4423
adj. R^2^	0.089	0.111	0.089	0.112
F value	4.325	18.301	4.680	18.352

Note: (1)***,** and * mark significance at the level of 1%, 5% and 10%,respectively; (2) Values in parenthesis are the t-values of the variables.

## Conclusions

This paper extends extant research by exploring the relationship between low-carbon pilot policy and urban economic resilience. Using the Heckman two stage model and the PSM-DID method, the paper empirically verifies that low-carbon pilot policy improves urban economic resilience in China. The low-carbon pilot policy is enhancing the economy’s capacity to resist, contain and dampen external shocks, suggesting a promising internal consistency of promoting economic stability and strengthening environmental protection.

This paper finds that the low-carbon pilot policy improves urban economic resilience by enhancing the growth of innovative technology. It shows that science and technology should never be overlooked as it is the underlying mechanism of how the good changes work. The technological mechanism is also consistent with the work of Yiwei and He [51], Zhou et al. [52] and Trembaczowski [45].

Moreover, this paper suggests that low-carbon pilot policy is more effective on improving the urban economic resilience of cities in central and western regions and non-first-tier cities, than the cities in eastern regions and first-tier cities. The geographical difference is consistent with observations in previous literature [53, 54].

This research may relieve concerns for policy makers wavering over whether to undertake low carbon transition initiatives such as low-carbon pilot policies. Firstly, this research empirically demonstrates that low-carbon pilot policy significantly improves urban economic resilience in China. Since China is a developing country, the policy experience may be applicable to other developing countries as well. Secondly, this research shows that the low-carbon city policy achieves more effective and advantageous outcomes in non-developed areas. Promoting low-carbon city policy will contribute to narrow the gap between developed and underdeveloped areas and reduce the disparities between them. Last but not least, establishing the mindset that low-carbon transition improves urban economic resilience will help policy makers to take carbon neutrality initiatives proactively.
